# Evaluation of the Relationship Between Neurologic Manifestations and Genetic Mutations in Wilson’s Disease with Next-Generation Sequencing

**DOI:** 10.3390/diagnostics15212689

**Published:** 2025-10-24

**Authors:** Sami Akbulut, Seyma Is, Tugba Kul Koprulu, Fatma Ilknur Varol, Zeynep Kucukakcali, Cemil Colak, Ahmet Koc, Saban Tekin, Sezai Yilmaz

**Affiliations:** 1 Department of Surgery and Liver Transplantation, Faculty of Medicine, Inonu University, 44280 Malatya, Türkiye; 2 Department of Biostatistics and Medical Informatics, Faculty of Medicine, Inonu University, 44280 Malatya, Türkiye; 3 Experimental Medicine Application and Research Center, University of Health Sciences, 34662 Istanbul, Türkiye; 4 Division of Bioinformatics, Department of Molecular Biotechnology, Faculty of Science, Turkish-German University, 34820 Istanbul, Türkiye; 5 Department of Molecular Medicine, Hamidiye Institute of Health Sciences, University of Health Sciences, 34668 Istanbul, Türkiye; 6 Department of Pediatric Gastroenterology, Hepatology and Nutrition, Faculty of Medicine, Inonu University, 44280 Malatya, Türkiye; 7 Department of Medical Biology and Genetics, Faculty of Medicine, Inonu University, 44280 Malatya, Türkiye; 8 Department of Basic Medical Sciences, Division of Medical Biology, Hamidiye Faculty of Medicine, University of Health Sciences, 34668 Istanbul, Türkiye

**Keywords:** Wilson’s disease, neurological Wilson’s disease, *ATP7B* protein, copper metabolism disorders, genetic variation, whole exome sequencing

## Abstract

**Background**: Wilson’s disease (WD) is a rare autosomal recessive disorder caused by mutations in the *ATP7B* gene, leading to copper accumulation in the liver and brain. Given the clinical heterogeneity of the disease, this study aimed to characterize the mutational spectrum of *ATP7B* and explore genotype–phenotype correlations in Turkish patients. **Methods**: Whole-exome sequencing (WES) was performed in 17 Turkish patients clinically diagnosed with WD. Variants were annotated and evaluated using five in silico prediction tools (REVEL, CADD, PolyPhen, SIFT, MutationTaster). Copy number variation (CNV) analysis was conducted using the CLC Genomics Server (Version 22.0.2). **Results**: A total of 14 distinct *ATP7B* variants were identified, comprising 12 missense, 1 nonsense, and 1 frameshift mutation. Variant distribution showed some phenotype-specific patterns: four variants were found more frequently in hepatic cases and three in neurological cases, although no statistically significant or consistent correlation between genotype and clinical presentation could be established. The most frequent mutation was p.His1069Gln, present in both phenotypes. All missense variants were predicted to be pathogenic by at least three computational tools, with high concordance among platforms. No pathogenic CNVs were detected. **Conclusions**: This study expands the mutational landscape of *ATP7B* in Turkish patients with WD and supports the utility of WES combined with in silico tools for accurate variant classification. The results emphasize the genetic heterogeneity of WD and suggest possible associations between certain mutations and clinical phenotypes.

## 1. Introduction

In 1912, Dr. Samuel Alexander Kinnier Wilson described a fatal condition involving progressive neurological impairment and liver cirrhosis—initially termed “progressive lenticular degeneration”—which later became known as WD [1,2]. WD is a rare autosomal-recessive monogenic disorder of copper metabolism caused by mutations in the *ATP7B* gene, which encodes a copper-transporting ATPase involved in both the incorporation of copper into caeruloplasmin and the excretion of excess copper; its dysfunction leads to disrupted copper homeostasis, resulting in toxic accumulation and progressive clinical feature [3,4,5,6]. Deficiency of the copper-transporting P1B-type ATPase *ATP7B* leads to pathological copper accumulation, primarily in the liver and secondarily in the central nervous system, resulting in a broad spectrum of clinical manifestations—from asymptomatic cases to acute liver failure [5,7,8].

The age-of-onset of WD typically ranges from 10 to 20 years, but cases have been reported as early as age 5 and as late as after 70; although most patients present early in life, WD should still be considered in middle-aged individuals, as hepatic symptoms tend to appear at younger ages, while neurologic presentations may occur later [1,9,10]. Earlier studies suggested that WD affects approximately 1 out of every 30,000 to 3 million individuals, with about 1 in 90 presumed to be carrier. More recent population-based molecular genetic analyses, however, indicate that WD may be considerably more common than previously assumed, with an estimated prevalence of approximately 1 in 7000 individuals [11,12,13]. Common features include highly variable symptoms such as progressive liver disease, as well as neurological, psychiatric, and ophthalmological symptoms [6].

Neurological symptoms of WD primarily involve extrapyramidal dysfunction and are highly variable, with dysarthria being the most common at presentation. Other frequent symptoms include gait abnormalities, dystonia (which may be focal, segmental, or generalized), tremor, parkinsonism, chorea, and less commonly, seizures. Characteristic signs include a dystonic facial expression known as risus sardonicus and the classic rubral “wing-beating” tremor, although tremors may also occur at rest, with posture, or during movement [1,2,14].

Hepatic symptoms in WD range from asymptomatic elevations in liver enzymes to fulminant liver failure. The disease typically begins with mild transaminitis, progressing to chronic hepatitis, fibrosis, and ultimately cirrhosis, which may advance from a compensated to a decompensated state with complications like ascites, coagulopathy, varices, and encephalopathy. Common presenting symptoms include jaundice, anorexia, and vomiting, followed by ascites and hepatosplenomegaly. Cirrhosis at diagnosis is linked to higher mortality risk, though hepatocellular carcinoma remains rare in WD [1,15,16,17].

More than 900 pathogenic mutations in the *ATP7B* gene have been identified in WD, with missense and nonsense single-nucleotide variants being the most common, followed by insertions, deletions, and splice-site mutations. The most frequent mutation, particularly among Caucasians, is p.H1069Q, accounting for 30–70% of cases and having an allelic frequency of 10–40% in the general population. Other recurrent mutations include p.E1064A, p.R778L, p.G943S, and p.M769V. Most patients are compound heterozygotes, carrying different mutations on each allele of the *ATP7B* gene. While many missense mutations result in complete loss of *ATP7B* function, protein-truncating mutations often lead to earlier disease onset due to decreased protein stability and quantity. Nevertheless, some variants retain partial copper-transporting activity, which may explain milder phenotypes.

Despite early findings suggesting a potential association between certain mutations—such as p.H1069Q—and specific clinical features like neurological symptoms, larger studies have failed to establish a consistent genotype–phenotype correlation. For instance, a cohort of 126 Bulgarian patients with high p.H1069Q frequency showed a wide range of hepatic and neurological presentations. Although some truncating mutations were associated with lower ceruloplasmin and copper levels, phenotypic variability remained high—even among individuals within the same family or monozygotic twins. This inconsistency points to the influence of genetic polymorphisms in *ATP7B*, variants in other genes, and epigenetic factors [3,18,19].

WD is diagnosed through a comprehensive approach that integrates clinical evaluation with biochemical and genetic testing. Diagnostic algorithms typically consider symptoms such as hepatic, neurological, or psychiatric manifestations, alongside tests assessing copper metabolism—namely reduced serum ceruloplasmin, elevated 24 h urinary copper excretion, hepatic copper quantification via liver biopsy, and molecular analysis of *ATP7B* gene mutations [20,21]. Early and accurate diagnosis is crucial, as timely treatment can prevent irreversible organ damage.

Management of WD requires lifelong therapy aimed at reducing copper accumulation. First-line treatments include copper-chelating agents such as D-penicillamine or trientine, which induce urinary excretion of copper, and zinc salts, which inhibit intestinal copper absorption. In advanced cases with hepatic decompensation or liver failure, liver transplantation remains as a therapeutic option [4,5,22]. Emerging treatments, including tetrathiomolybdate salts and gene therapy, are under investigation and may offer future alternatives.

Although the molecular basis of *ATP7B* dysfunction is well established, clinical heterogeneity—particularly the variable presence of neurological and psychiatric symptoms—remains poorly understood. Despite numerous studies, a clear genotype-phenotype correlation has yet to be defined. This study aims to further explore these correlations to improve prediction of clinical outcomes and personalize treatment strategies in WD.

## 2. Materials and Methods

### 2.1. Sample Collection

The present study enrolled a total of 30 participants. Among them, 17 were patients with a confirmed diagnosis of WD: 10 exhibited predominantly neurological manifestations, whereas seven presented with typical hepatic involvement. Three additional individuals initially displayed WD-like clinical symptoms but were later excluded from the WD diagnosis after comprehensive evaluation, serving as a disease-mimic comparison group. In addition, 10 age- and sex-matched healthy controls were included. All patients were routinely followed up at the Department of Pediatric Hepatology, Liver Transplantation Institute, Inonu University Faculty of Medicine, where diagnoses were established through a combination of clinical evaluation, comprehensive biochemical assessment, and, when indicated, histopathological confirmation. All patients enrolled in this study had received their diagnosis during childhood, which was determined according to the scoring system proposed by Ferenci et al. [23]. The control group consisted of children without chronic illnesses who presented to the pediatric outpatient clinic with self-limited conditions, such as mild upper respiratory tract infections or nonspecific pain complaints, ensuring minimal confounding factors. Peripheral blood samples were obtained from all participants using standardized PAXgene Blood DNA Tubes (Zymo Research, Irven, CA, USA), processed under uniform laboratory conditions. Written and verbal informed consent was obtained from parents or legal guardians prior to participation, and the protocol was reviewed and approved by the Malatya Clinical Research Ethics Committee (Approval Number: 99, Date: 28 September 2022)

### 2.2. DNA Isolation

DNA isolation was performed at the University of Health Sciences Experimental Medicine Application and Research Center under standardized laboratory conditions to minimize pre-analytical variability. Genomic DNA (gDNA) was extracted from whole blood samples using the chemagic DNA Blood protocol on the fully automated Chemagic 360 DNA/RNA extraction platform (Perkin Elmer, Waltham, MA, USA) according to the manufacturer’s guidelines. DNA yield and purity were quantified using a Qubit 4.0 fluorometer (Invitrogen, Waltham, MA,, USA) and validated with Take3 microvolume plates on the BioTek Synergy Neo2 Multimode Reader (Agilent Technologies, Santa Clara, CA, USA) for cross-checking. The resulting gDNA concentrations ranged from 11.4 to 64.8 ng/µL, with OD260/280 ratios consistently between 1.8 and 2.0, confirming high purity suitable for downstream sequencing. All samples were aliquoted and stored at +4 °C until subsequent analysis to preserve integrity.

### 2.3. Library Preparation and WES

Library preparation and subsequent WES analysis were performed at the University of Health Sciences Experimental Medicine Application and Research Center under rigorously standardized conditions to ensure reproducibility. WES libraries were prepared using the QIAseq Human Exome Kit (Qiagen GmbH, Hilden, Germany) with 50 ng of high-quality input gDNA, strictly following the manufacturer’s recommendations. In brief, genomic DNA was enzymatically fragmented with the QIAseq FX DNA Library Kit (Qiagen GmbH, Hilden, Germany), and fragment ends were repaired to yield blunt, 5′-phosphorylated fragments. A-tailing was subsequently performed to facilitate efficient adapter ligation. Each fragment was ligated with uniquely barcoded adapters, enabling sample multiplexing, and PCR amplification was performed to generate sufficient material for capture. Six indexed libraries were equimolarly pooled and hybridized with biotinylated capture probes to enrich for exonic regions. Captured fragments were retrieved using streptavidin-coated magnetic beads, while off-target DNA was removed by stringent sequential washing. A second round of PCR amplification was applied to increase library yield. Library quantity and quality were confirmed with the Qubit dsDNA High Sensitivity Assay on Qubit 4.0 fluorometer (Invitrogen, Waltham, MA, USA) and size distribution was analyzed using the D1000 ScreenTape Assay on a TapeStation 4150 (Agilent Technologies, Santa Clara, CA, USA). Final qualified libraries were sequenced on the Illumina NovaSeq 6000 platform (Illumina, San Diego, CA, USA) with paired-end reads (2 × 150 bp) using the Pool and Denature Libraries for Sequencing (Standard Loading) protocol, generating high-depth coverage suitable for downstream variant calling.

### 2.4. Analysis of WES Raw Data and Variant Interpretation

WES raw data analysis was conducted for 19 patients using the CLC Genomics Server (Version 22.0.2) [24] after converting BCL files to FASTQ format. Sequence alignment was performed against the human reference genome hg19 (GRCh37). Duplicate reads were removed using the Remove Duplicate Mapped Reads tool in QIAGEN CLC Genomics Workbench (Version 22.0.2). Variant calling was carried out with the following parameters: a minimum coverage threshold of 5×, a minimum variant allele frequency of 20%, and an average base quality score of Q30. Annotation was based on the Ensembl gene and transcript annotation (release 87) based on the GRCh37 (hg19) reference genome. Additional datasets used for variant annotation included PhastCons conservation scores for hg19, the 1000 Genomes Project Phase 3 data (Ensembl v106 hg19), and dbSNP common variants (v151 Ensembl hg19).

For variant interpretation, the QIAGEN Clinical Insight Interpret (QCI-I) software (Version 9.2.1.20231012) was used. Variants that met the quality criteria (Q30 or higher) were classified according to the Human Genome Variation Society (HGVS) nomenclature and the American College of Medical Genetics and Genomics (ACMG) guidelines. The Integrative Genomics Viewer (IGV) was used to visualize BAM files and assess potential sequencing artifacts. Tertiary analysis and clinical interpretation of the variants were performed using QCI-I software. Subsequent filtering steps, following the IGV visualization, enabled the identification of potentially causative variants.

### 2.5. Detection of CNVs Using Control Data from WES

A group of ten control samples known to carry non-pathogenic CNVs was sequenced using the same WES protocol described above. These control samples served as a baseline for comparison with patient data. Pathogenic CNVs were identified using the CNV Detection Tool in CLC Genomics Server (Version 22.0.2).

### 2.6. Pathogenicity Prediction of Identified Variants

To evaluate the potential damaging effects of the identified variants, we employed multiple computational prediction tools. Specifically, in silico pathogenicity assessments were conducted using Rare Exome Variant Ensemble Learner (REVEL) (v1.3) [25], Combined Annotation-Dependent Depletion (CADD) (v1.6) [26], PolyPhen (v2.2.2) [27], Sorting Intolerant From Tolerant (SIFT) (v6.2.1) [28], and MutationTaster (v2021) [29] software.

## 3. Results

### 3.1. Identification of Variants and CNVs

**Figure 1:**
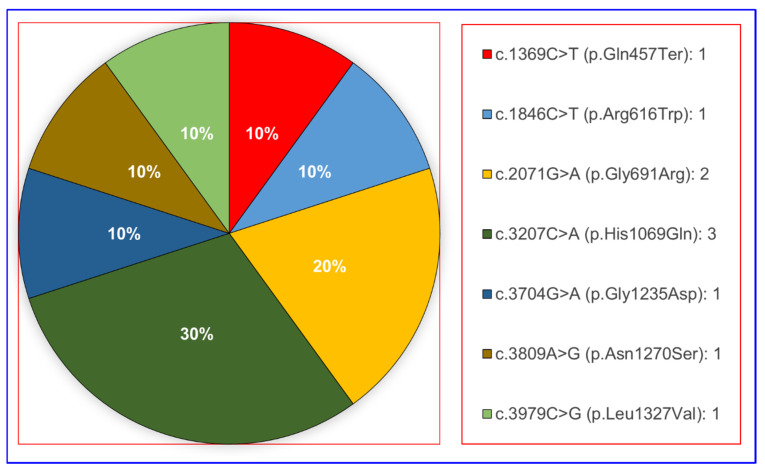
Distribution of detected variants in WD patients with hepatic symptoms alone.

**Figure 2:**
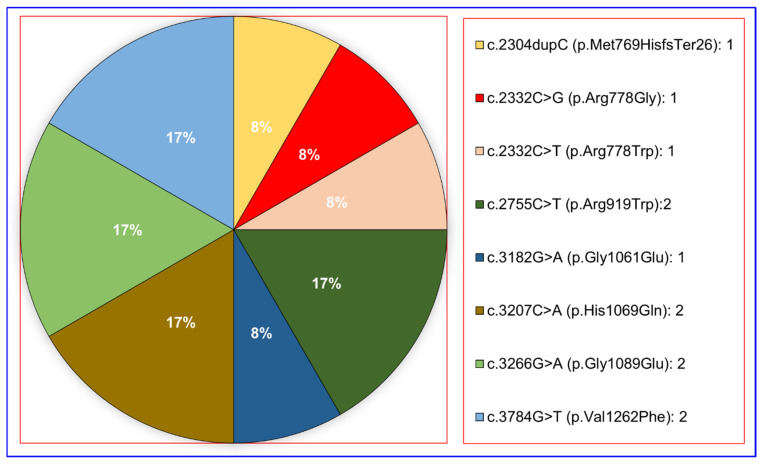
Distribution of detected variants in WD patients with neurological symptoms.

WES analysis identified 14 unique variants of the *ATP7B* protein in 17 Turkish patients. Seven mutations were detected in four WD patients with hepatic symptoms alone and 12 mutations (eight mutations when deduplicated) in 10 WD patients with neurological symptoms (Figure 1 and Figure 2).

**Figure 3:**
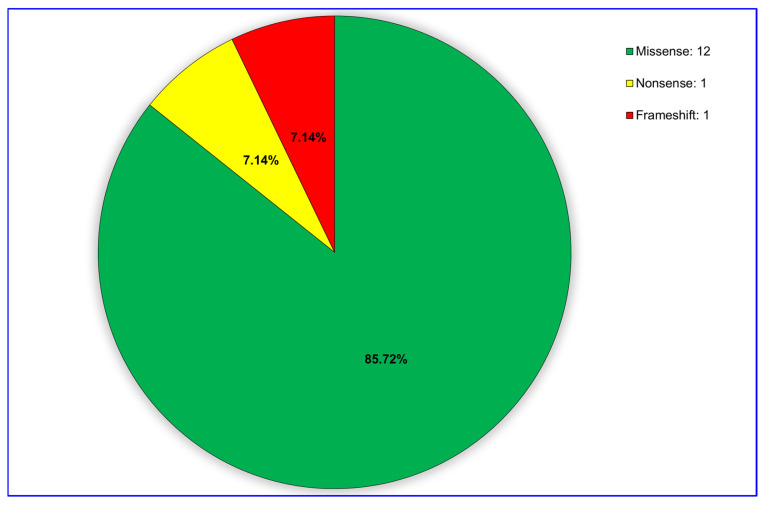
Distribution of detected variants regarding the effect on *ATP7B* protein.

Regarding the mutation types, WES analysis detected 12 missense mutations, 1 nonsense mutation, and 1 frameshift mutation (Figure 2). Missense variants were detected as c.1846C > T (p.Arg616Trp), c.2071G > A (p.Gly691Arg), c.2332C > G (p.Arg778Gly), c.2332C > T (p.Arg778Trp), c.2755C > T (p.Arg919Trp), c.3182G > A (p.Gly1061Glu), c.3207C > A (p.His1069Gln), c.3266G > A (p.Gly1089Glu), c.3704G > A (p.Gly1235Asp), c.3784G > T (p.Val1262Phe), c.3809A > G (p.Asn1270Ser), and c.3979C > G (p.Leu1327Val); while the nonsense variant was identified as c.1369C > T (p.Gln457Ter) and the frameshift mutation as c.2304dupC (p.Met769HisfsTer26) (Figure 3 and Table 1).

In our data, the variants c.1369C > T (p.Gln457Ter), c.1846C > T (p.Arg616Trp), c.2071G > A (p.Gly691Arg), c.3207C > A (p.His1069Gln), 3704G > A (p.Gly1235Asp), c.3809A > G (p.Asn1270Ser), and c.3979C > G (p.Leu1327Val) were predominantly found in WD patients with hepatic symptoms. On the contrary, c.2332C > G (p.Arg778Gly), c.2332C > T (p.Arg778Trp), and c.3182G > A (p.Gly1061Glu) were more frequently in WD patients with neurological symptoms. The missense variant c.3207C > A (p.His1069Gln) and the nonsense variant c.1369C > T (p.Gln457Ter) were identified in both WD with hepatic symptoms and WD with neurological symptoms.

All variants except c.2071G > A (p.Gly691Arg), c.2304dupC (p.Met769HisfsTer26), c.2332C > G (p.Arg778Gly), and c.2332C > T (p.Arg778Trp) are located in the topological domain of the *ATP7B* protein, while the latter four affect the transmembrane domain of *ATP7B*.

WES did not reveal any pathogenic CNVs in the patient samples. Ten control samples with known non-pathogenic CNVs were sequenced in parallel using the same protocol and analyzed for comparison. Using the CNV detection tool in CLC Genomics Server (Version 22.0.2), no pathogenic CNVs were detected in any samples. Nevertheless, due to the limited sensitivity of WES for certain CNV types, clinically relevant variants cannot be completely ruled out.

### 3.2. Prediction of the Pathogenicity of Variants and Genotype-Phenotype Correlations

The functional predictions for 11 missense variants in the *ATP7B* gene are given in Table 2, assessed using five widely accepted computational tools: REVEL, CADD, PolyPhen, SIFT, and MutationTaster. These tools collectively evaluate the potential deleteriousness of each variant based on evolutionary conservation, biochemical properties, and predicted impact on protein structure and function.

All tested variants were classified as deleterious by REVEL, with scores ranging from 0.75 to 0.975, exceeding the commonly used threshold of 0.5 that suggests pathogenicity. CADD scores for all variants were above 23.0, with some reaching as high as 32.0, indicating a high likelihood of functional impact (CADD scores >20 are considered potentially pathogenic). According to PolyPhen, all but one variant (p.Leu1327Val) were predicted to be probably damaging, while the latter was classified as possibly damaging. SIFT predicted damaging effects for all variants except p.Gly1089Glu, which was classified as tolerated. MutationTaster consistently predicted all variants to be disease-causing, supporting their potential pathogenic role in WD (Table 2).

Overall, the high concordance among different prediction tools strongly suggests that these variants likely affect *ATP7B* protein function. Notably, well-known pathogenic mutations such as p.His1069Gln, p.Arg778Trp, and p.Gly1061Glu showed particularly high REVEL and CADD scores, consistent with their established clinical significance. The consistent classification of most variants as deleterious or disease-causing supports their potential relevance in disease etiology and underscores the utility of integrating multiple computational prediction platforms in variant assessment.

In cases H1 and N4, a homozygous missense variant, c.3979C > G; p.Leu1327Val, in *ATP7B* (NM_000053.4) was identified. This p.Leu1327Val mutation occurs within the HAD-like domains of the *ATP7B* protein. Predicted to result in a loss of function, the alteration is deemed deleterious within exon 19 of the NM_000053.4 transcript. Although this missense variant was absent in gnomAD v4.1, it is documented in HGMD under the accession ID CM993113 and classified as a “Disease Mutation (DM)”. The p.Leu1327Val alteration has been previously reported in two distinct publications [30,31].

A homozygous c.2332C > T; p.Arg778Trp missense variant in *ATP7B* (NM_000053.4) was identified in case H2 and N3. The p.Arg778Trp mutation occurs within the HAD-like domains of *ATP7B*. This homozygous variant is predicted to cause a loss of function, being deleterious in exon 19 of the NM_000053.4 transcript. The p.Leu1327Val missense variant was observed in gnomAD v4.1 with an allele frequency of 0.00007125, and is recorded in HGMD under the CM970140 accession ID, classified as a “Disease Mutation (DM)”. The p.Arg778Trp alteration has been reported in eight different publications [31,32,33,34,35,36,37,38].

Case H3 presents two heterozygous missense variants in *ATP7B* (NM_000053.4): c.1846C > T; p.Arg616Trp and c.3809A > G; p.Asn1270Ser. The first variant, p.Arg616Trp, occurs within the HMA domains of *ATP7B*. This alteration is classified as a compound heterozygous loss of function due to the presence of another deleterious variant in the same *ATP7B* transcript (NM_000053.4). The p.Arg616Trp variant was found in gnomAD v4.1 with an allele frequency of 0.00001673, recorded in HGMD with the CM014320 accession ID, and classified as a “Disease Mutation (DM)”. This variant has been reported in six different publications [38,39,40,41,42,43].

The second variant of case H3, p.Asn1270Ser, occurs within the HAD-like domains of *ATP7B*. It is functionally characterized as a known loss of function in the literature. The p.Asn1270Ser variant was observed in gnomAD v4.1 with an allele frequency of 0.0001431, recorded in HGMD with the CM930060 accession ID, and also classified as a “Disease Mutation (DM)”. This variant has been reported in twenty-six different publications [31,32,33,36,38,41,42,44,45,46,47,48,49,50,51,52,53,54,55,56,57,58,59,60,61,62].

A homozygous c.3207C > A; p.His1069Gln missense variant in *ATP7B* (NM_000053.4) was identified in cases H4, N1, and N4. The p.His1069Gln mutation occurs within the HAD-like domains of *ATP7B* and is functionally recognized as a loss of function in the literature. This variant was observed in gnomAD v4.1 with an allele frequency of 0.001019, recorded in HGMD under the CM930059 accession ID, and classified as a “Disease Mutation (DM)”. The p.His1069Gln alteration has been reported in forty-six publications [31,32,34,36,41,42,46,50,54,59,63,64,65,66,67,68,69,70,71,72,73,74,75,76,77,78,79,80,81,82,83,84,85,86,87,88,89,90,91,92,93,94,95,96,97].

Two heterozygous variants in *ATP7B* (NM_000053.4) were identified in case H5: c.1369C > T; p.Gln457Ter and c.3704G > A; p.Gly1235Asp. The first variant, p.Gln457Ter, is a stop gain mutation located outside of the characterized functional domains and does not correspond to benign variation. This variant is classified as a compound heterozygous loss of function, as it is accompanied by another deleterious variant in the same *ATP7B* transcript (NM_000053.4). The p.Gln457Ter nonsense variant was not found in gnomAD v4.1, but it is recorded in HGMD under the CM992591 accession ID and classified as a “Disease Mutation (DM)”. This alteration has been reported in three different publications [50,98,99].

The second variant of case H5, p.Gly1235Asp, is a missense mutation located within the HAD-like domains. It is functionally characterized as a known loss of function in the literature. The p.Gly1235Asp variant was not observed in gnomAD v4.1 but is listed in HGMD with the CM1510476 accession ID and classified as a “Disease Mutation (DM)”. This missense alteration has been reported in two different publications [42,100].

In cases H6 and H7, a homozygous missense variant c.2071G > A (p.Gly691Arg) in the *ATP7B* gene (NM_000053.4) was identified. This alteration occurs within the HAD-like domains and is predicted to result in loss of function, as it is considered deleterious in exon 7 of the transcript. The p.Leu1327Val missense variant, although absent from gnomAD v4.1, is recorded in the Human Gene Mutation Database (HGMD) under accession number CM980170 and is classified as a “Disease Mutation (DM).” It has been reported in six independent publications [31,32,50,101,102,103].

A homozygous c.3182G > A; p.Gly1061Glu missense variant in *ATP7B* (NM_000053.4) was identified in case N2. The p.Gly1061Glu missense mutation, located within the HAD-like domains of *ATP7B*, is predicted to cause a loss of function, being deleterious in exon 7 of the NM_000053.4 transcript. This variant was not observed in gnomAD v4.1 but is recorded in HGMD under the CM980170 accession ID and classified as a “Disease Mutation (DM)”. The p.Gly1061Glu alteration has been reported in six different publications [31,32,52,98,104,105].

In case N5, a homozygous c.2332C > G; p.Arg778Gly missense variant in *ATP7B* (NM_000053.4) was detected. This p.Arg778Gly mutation, which resides within the HAD-like domains of *ATP7B*, is predicted to result in a loss of function due to its deleterious effect in exon 8 of the NM_000053.4 transcript. The variant was found in gnomAD v4.1 with an allele frequency of 0.00001177, listed in HGMD under the CM950112 accession ID, and classified as a “Disease Mutation (DM)”. This missense alteration has been documented in ten publications [31,34,36,42,50,73,95,106,107].

A homozygous c.2755C > T; p.Arg919Trp missense variant in *ATP7B* (NM_000053.4) was identified in cases N6 and N7. The p.Arg919Trp mutation, located within the HAD-like domains of *ATP7B*, is predicted to result in a loss of function due to its deleterious effect in exon 12 of the NM_000053.4 transcript. This variant was observed in gnomAD v4.1 with an allele frequency of 0.00003222, recorded in HGMD under the CM980176 accession ID, and classified as a “Disease Mutation (DM)”. The p.Arg919Trp alteration has been documented in five different publications [31,33,48,77,103].

In case N8, a homozygous c.2304dupC; p.Met769HisfsTer26 frameshift variant in *ATP7B* (NM_000053.4) was identified. This p.Met769HisfsTer26 mutation, located within the HAD-like domains, is predicted to result in a loss of function due to its harmful impact in exon 8 of the NM_000053.4 transcript. The variant was observed in gnomAD v4.1 with an allele frequency of 0.00009789, listed in HGMD under the CI951903 accession ID, and classified as a “Disease Mutation (DM)”. This frameshift alteration has been reported in twenty-four publications [32,33,38,42,43,46,48,49,50,53,57,65,77,89,108,109,110,111,112,113,114,115,116,117].

Two heterozygous variants in *ATP7B* (NM_000053.4) were identified in cases N9 and N10: c.3266G > A; p.Gly1089Glu and c.3784G > T; p.Val1262Phe. The first variant, p.Gly1089Glu, is a missense mutation located within the HAD-like domains. This variant is considered a compound heterozygous loss of function due to the presence of another deleterious variant in the same *ATP7B* transcript (NM_000053.4). The p.Gly1089Glu variant was not observed in gnomAD v4.1 but is recorded in HGMD under the CM960130 accession ID and classified as a “Disease Mutation (DM)”. This alteration has been reported in three publications [31,42,118].

The second variant, p.Val1262Phe, is a missense mutation within the HAD-like domains, also considered a compound heterozygous loss of function due to the presence of another deleterious variant in the same transcript. The p.Val1262Phe variant was observed in gnomAD v4.1 with an allele frequency of 0.000005576, recorded in HGMD with the CM992828 accession ID, and classified as a “Disease Mutation (DM)”. This alteration has been reported in four different publications [30,36,93].

Among the variants observed, p.His1069Gln (exon 11) was the most frequent, found in multiple cases (cases H4, N1, and N4) and associated with both hepatic and neurological manifestations. The allele frequency of p.His1069Gln in gnomAD v4.1 was 0.1019%, suggesting it is relatively common in the population (Table 3).

Rare variants included p.Arg616Trp (exon 8) with an allele frequency of 0.001673%, p.Arg778Trp (exon 19) with 0.007125%, p.Arg919Trp (exon 12) with 0.003222%, p.Met769HisfsTer26 (exon 8) with 0.009789, p.Val1262Phe (exon 13) with 0.0005576%, and likely p.Leu1327Val (exon 19), p.Gln457Ter (exon 5), p.Gly1235Asp (exon 12), p.Gly1061Glu (exon 14), and p.Gly1089Glu (exon 13) with no available allele frequency in gnomAD v4.1.

The variants p.Leu1327Val (exon 19), p.Arg616Trp (exon 8), p.Asn1270Ser (exon 18), p.Gln457Ter (exon 5), and p.Gly1235Asp (exon 12) were observed in individuals with hepatic symptoms, suggesting a preliminary association that warrants further investigation, although a causal relationship cannot be established based on the available data.

Several mutations, namely p.Gly1061Glu (exon 14), p.Arg778Gly (exon 19), p.Arg919Trp (exon 12), p.Met769HisfsTer26 (exon 8), p.Gly1089Glu (exon 13), and p.Val1262Phe (exon 13) were identified only in patients with neurological symptoms, all being extremely rare in the general population. Overall, the allele frequencies and mutation types observed provide insights into the genetic basis of WD, with some variants showing a higher likelihood of contributing to either hepatic or neurological symptoms.

Despite our extensive analysis, no clear or significant correlation between genotype and phenotype was observed in the data. The genetic variants detected in the *ATP7B* gene did not exhibit a consistent relationship with the clinical manifestations of WD across the individuals studied. This absence of correlation, though unexpected, suggests that other factors—possibly environmental, epigenetic, or yet unidentified genetic influences—may play a role in shaping the clinical presentation of the disease. Further investigation, involving larger cohorts or additional genetic and clinical data, will be necessary to unravel the complexities of genotype-phenotype interactions in WD.

Demographic data indicated that the mean age was 12.78 years in WD patients with hepatic symptoms and 18.1 years in WD patients with neurological symptoms. Four of the seven WD patients with hepatic symptoms and five of the 10 WD patients with neurological symptoms were males (Table 4).

Biochemical parameters indicated that case N2 with the variation c.3182G > A (p.Gly1061Glu) had the highest aspartate aminotransferase (AST) and alanine aminotransferase (ALT) levels, indicating hepatocellular injury. Furthermore, case N2 also had an elevated creatinine (CRE) level, revealing severe liver failure. This phenotype was associated with c.3182G > A (p.Gly1061Glu) in exon 14, which was previously also found in patients with liver diseases explaining the severe phenotype of case N2 [119].

Cases N9 and N10, who are siblings, were found to carry identical mutations in the *ATP7B* gene. Despite this shared genotype, their clinical and biochemical profiles differ markedly. Notably, case N10 presented with a Kayser-Fleischer ring, whereas case N9 did not. In terms of liver enzymes, alkaline phosphatase (ALP) and gamma-glutamyl transferase (GGT) levels were higher in case N9 compared to case N10. Conversely, total bilirubin levels were substantially lower in case N9. Furthermore, 24 h urinary copper excretion was more than twice as high in case N9 as in case N10. These intra-familial phenotypic differences, despite identical genotypes, highlight the variability in clinical presentation of WD.

This case pair clearly illustrates one of the major challenges in WD research: the lack of a consistent genotype–phenotype correlation. Even among individuals with the same pathogenic variants and shared environmental background, clinical manifestations can vary significantly. Such findings emphasize the influence of yet unidentified genetic modifiers, epigenetic factors, or other external variables, and explain why attempts to establish a straightforward correlation between genotype and phenotype in WD have so far been inconclusive.

## 4. Discussion

In this study, we identified several genetic variants in the *ATP7B* gene in 17 Turkish patients with WD, which were associated with either hepatic or neurological manifestations. We observed 14 unique variants, including 12 missense mutations, 1 nonsense mutation, and 1 frameshift mutation. Interestingly, certain variants such as p.His1069Gln (exon 11) were common across both hepatic and neurological manifestations, while others, like p.Gly1061Glu (exon 14), p.Arg778Trp (exon 19), p.Arg778Gly (exon 19), p.Arg919Trp (exon 12), p.Met769HisfsTer26 (exon 8), p.Gly1089Glu (exon 13), and p.Val1262Phe (exon 13), were predominantly linked to neurological symptoms in our cohort. Despite these findings, no clear or significant genotype-phenotype correlation was observed between the detected *ATP7B* variants and the clinical features of the disease.

Although the *ATP7B* gene mutations identified were predicted to be pathogenic based on various computational tools, including REVEL, CADD, PolyPhen, and SIFT, we were unable to establish a consistent genotype-phenotype relationship in the patients studied. The prediction tools indicated that most of the variants, including well-known pathogenic mutations such as p.His1069Gln, p.Arg616Trp, and p.Gly1061Glu, were likely to cause a loss of function, suggesting their potential contribution to the clinical manifestations of WD. However, despite the identification of several rare variants, there was no direct correlation between the presence of these mutations and the severity or type of symptoms observed, specifically between hepatic and neurological involvement.

This lack of correlation aligns with the known clinical heterogeneity of WD, where individuals with identical mutations can exhibit a range of symptoms. Several studies have documented similar discrepancies, where patients with the same *ATP7B* mutation may present with either severe hepatic disease or neurological symptoms, or both, without a clear genetic explanation for the differential phenotype expression [120,121,122].

The absence of a clear genotype-phenotype correlation in this cohort might be attributed to modifying factors such as epigenetics, environmental influences, or genetic modifiers. For instance, epigenetic mechanisms—such as DNA methylation or histone modifications—could modulate the expression of the *ATP7B* gene, influencing copper metabolism and the onset of symptoms. Furthermore, environmental factors, such as diet and exposure to oxidative stress, may affect copper accumulation and exacerbate symptoms, especially in the liver or brain.

Another critical factor is the possibility of genetic modifiers, which could include other genes involved in copper transport, metabolism, or detoxification. Studies have suggested that *ATP7B* mutations alone may not fully account for the observed phenotypic diversity in WD. For example, mutations in other copper-transporting genes or genes related to neurodegeneration could interact with *ATP7B* defects and influence the disease’s clinical course.

The case of siblings (cases N9 and N10), who share identical mutations in the *ATP7B* gene, highlights the complex and variable nature of WD. Despite the same genetic background, these siblings displayed significantly different clinical profiles. Notably, case N10 developed a Kayser-Fleischer ring, a hallmark sign of WD, while case N9 did not. Biochemical data further revealed that case N9 had higher levels of liver enzymes, including alkaline phosphatase (ALP) and gamma-glutamyl transferase (GGT), while case N10 exhibited higher total bilirubin levels. The most striking difference was in 24 h urinary copper excretion, which was more than twice as high in case N9 compared to case N10. These findings strongly suggest that factors beyond the *ATP7B* gene—such as other genetic variations, environmental influences, or disease-modifying factors—play a significant role in shaping the clinical manifestation of WD in genetically identical individuals.

The phenotypic divergence observed between cases N9 and N10, despite their identical *ATP7B* mutations, underscores the complexity of genotype–phenotype correlations in WD. Such intra-familial variability highlights the need for individualized clinical evaluation, even among patients sharing the same genetic background, and supports the broader notion that the clinical spectrum of WD cannot be fully predicted by genotype alone.

Such intra-familial differences are commonly observed in WD and illustrate one of the primary challenges in understanding the genotype-phenotype correlation in this disorder. Despite identical genetic mutations, clinical outcomes can vary significantly, underscoring the influence of genetic modifiers and other unidentified factors on the disease’s progression and manifestation.

One of the main limitations of our study is the small sample size (17 patients), which limits the power of statistical analyses to detect genotype-phenotype associations. Larger cohort studies with more comprehensive clinical and genetic data are needed to explore the potential role of genetic modifiers and environmental factors in shaping the clinical presentation of WD. Additionally, further research on the functional impact of rare and novel *ATP7B* mutations, especially those without known allele frequencies in gnomAD v4.1, could provide more insights into their role in disease pathogenesis.

Our study is the first to investigate *ATP7B* gene mutations in the eastern region of Türkiye, adding to the growing body of knowledge regarding WD in the country. To date, there have been three studies conducted on *ATP7B* mutations in Türkiye, with research centers based in the cities Ankara and İzmir [123,124,125] and one study involving Turkish WD patients among other nationalities [118]. These studies have led to the identification of various genetic variants associated with WD, contributing to our understanding of its genetic landscape. However, in our study, we have identified several variants that have never been reported before in any of these previous studies, marking the first observation of these variants in the Turkish population. These novel variants are listed as follows: c.1846C > T (p.Arg616Trp) (exon 8), c.3704G > A (p.Gly1235Asp) (exon 12), c.3182G > A (p.Gly1061Glu) (exon 14), c.3266G > A (p.Gly1089Glu) (exon 13), c.3784G > T (p.Val1262Phe) (exon 13).

Despite the limited sample size of 17 patients, our study contributes valuable insights into the *ATP7B* variant spectrum in the Turkish population. Although the detection of a relatively high number of distinct variants is noteworthy, such diversity within a small cohort does not permit definitive conclusions regarding genotype-phenotype correlations. Rather, these findings expand the existing catalog of *ATP7B* variants and emphasize the considerable genetic heterogeneity of WD in Türkiye. The results highlight the importance of population-specific genetic studies and underscore the need for comprehensive mutation screening strategies to improve diagnostic accuracy in Turkish individuals with WD.

## 5. Conclusions

This study identified 14 distinct *ATP7B* gene variants, including 12 missense, 1 nonsense, and 1 frameshift mutation, in 17 Turkish patients with WD. Although some variants appeared more frequently in one phenotype, with certain mutations occurring predominantly in patients with hepatic or neurological symptoms, no statistically significant or consistent correlations between genotype and clinical presentation could be established. The most frequent mutation in this cohort was p.His1069Gln, detected in both clinical subtypes, consistent with its well-established role in WD pathogenesis. All missense variants were predicted to be pathogenic by multiple in silico tools, underscoring their potential functional relevance. The absence of pathogenic CNVs suggests that single-nucleotide variants and small indels are the predominant genetic alterations in this cohort. These findings highlight the genetic heterogeneity of *ATP7B* mutations in Turkish patients with WD.

In conclusion, while the *ATP7B* gene variants identified in our cohort are predicted to be pathogenic, the lack of consistent genotype-phenotype correlation in WD suggests that other factors—likely genetic modifiers, epigenetic influences, and environmental factors—are crucial in determining disease outcomes. The intra-familial differences observed in cases N9 and N10 further emphasize the complexity of WD and highlight the need for larger, more detailed studies to unravel the genetic and environmental factors contributing to the clinical heterogeneity of this disorder.

Although our study included only 17 patients, which is a small sample size, it is important to consider that WD is a rare genetic disorder. Despite the small cohort, we identified a striking diversity of *ATP7B* variants, underscoring the rich genetic landscape of WD in the Turkish population. This observation is consistent with previous reports that the molecular genetic spectrum of the *ATP7B* gene can vary across populations. Notably, several variants identified in our study—namely c.1846C > T, c.2071G > A, c.2332C > T, c.2755C > T, c.3182G > A, c.3266G > A, c.3704G > A, and c.3784G > T—were not observed in the Turkish cohorts from Ankara and İzmir, further highlighting regional differences in the mutational landscape [123,124,125]. Thus, the variants detected in our cohort further contribute to characterizing the molecular profile of WD specifically within the Turkish population. This highlights the value of conducting genetic studies even with limited sample sizes, as such data contribute essential insights for future meta-analyses and population-specific diagnostic strategies. Given the rarity of the disease, each patient adds valuable information to our understanding of its genetic diversity and clinical variability.

## Figures and Tables

**Table 1:** Details of identified *ATP7B* variants based on phenotype, cDNA and protein nomenclature, zygosity, protein domain, and HGMD accession ID.

Phenotype	Case ID	Mutation (cDNA)	Mutation (Protein)	Zygosity	Mutation Type	Protein Domain	Description	HGMD Accession ID
**WD with Hepatic Symptoms**	H1	c.3979C > G	p.Leu1327Val	Homozygous	Missense	Topological	Cytoplasmic	CM993113 (DM)
	H2	c.2332C > T	p.Arg778Trp	Homozygous	Missense	Transmembrane	Helical	CM970140 (DM)
	H3	c.1846C > T	p.Arg616Trp	Heterozygous	Missense	Topological	Cytoplasmic	CM014320 (DM)
		c.3809A > G	p.Asn1270Ser	Heterozygous	Missense	Topological	Cytoplasmic	CM930060 (DM)
	H4	c.3207C > A	p.His1069Gln	Homozygous	Missense	Topological	Cytoplasmic	CM930059 (DM)
	H5	c.1369C > T	p.Gln457Ter	Heterozygous	Nonsense	Topological	Cytoplasmic	CM992591 (DM)
		c.3704G > A	p.Gly1235Asp	Heterozygous	Missense	Topological	Cytoplasmic	CM1510476 (DM)
	H6	c.2071G > A	p.Gly691Arg	Homozygous	Missense	Transmembrane	Helical	CM980170 (DM)
	H7	c.2071G > A	p.Gly691Arg	Homozygous	Missense	Transmembrane	Helical	CM980170 (DM)
**WD with Neurological Symptoms**	N1	c.3207C > A	p.His1069Gln	Homozygous	Missense	Topological	Cytoplasmic	CM930059 (DM)
	N2	c.3182G > A	p.Gly1061Glu	Homozygous	Missense	Topological	Cytoplasmic	CM992600 (DM)
	N3	c.2332C > T	p.Arg778Trp	Homozygous	Missense	Transmembrane	Helical	CM970140 (DM)
	N4	c.3207C > A	p.His1069Gln	Homozygous	Missense	Topological	Cytoplasmic	CM930059 (DM)
	N5	c.2332C > G	p.Arg778Gly	Homozygous	Missense	Transmembrane	Helical	CM950112 (DM)
	N6	c.2755C > T	p.Arg919Trp	Homozygous	Missense	Topological	Cytoplasmic	CM980176 (DM)
	N7	c.2755C > T	p.Arg919Trp	Homozygous	Missense	Topological	Cytoplasmic	CM980176 (DM)
	N8	c.2304dupC	p.Met769HisfsTer26	Homozygous	Frameshift	Transmembrane	Helical	CI951903 (DM)
	N9	c.3266G > A	p.Gly1089Glu	Heterozygous	Missense	Topological	Cytoplasmic	CM960130 (DM)
		c.3784G > T	p.Val1262Phe	Heterozygous	Missense	Topological	Cytoplasmic	CM992828 (DM)
	N10	c.3266G > A	p.Gly1089Glu	Heterozygous	Missense	Topological	Cytoplasmic	CM960130 (DM)
		c.3784G > T	p.Val1262Phe	Heterozygous	Missense	Topological	Cytoplasmic	CM992828 (DM)

**Table 2:** Pathogenicity predictions of missense mutations were obtained using REVEL, CADD, PolyPhen, SIFT, and MutationTaster. REVEL scores range from 0 to 1, with scores >0.5 indicating ‘likely disease causing’ and <0.5 ‘likely benign’ variants. CADD scores >30 are considered as ‘likely deleterious’ and scores <30 as ‘likely benign’. PolyPhen scores >0.908 are classified as ‘probably damaging’, scores between 0.446–0.908 as ‘possibly damaging’, and scores ≤0.446 as ‘benign’. SIFT scores <0.05 are defined as ‘deleterious’, whereas scores >0.05 are defined as ‘tolerated’. MutationTaster provides qualitative predictions: “disease causing” (‘likely deleterious’) or “polymorphism” (‘likely benign’), with confidence levels based on supporting evidence.

*ATP7B* Variant	REVEL	CADD	PolyPhen	SIFT	Mutation Taster
c.3979C > G; p.Leu1327Val	Deleterious (Score: 0.793)	Deleterious (Score: 24.9)	Possibly Damaging	Damaging	Disease Causing
c.2332C > T; p.Arg778Trp	Deleterious (Score: 0.877)	Deleterious (Score: 25.4)	Probably Damaging	Damaging	Disease Causing
c.1846C > T; p.Arg616Trp	Deleterious (Score: 0.813)	Deleterious (Score: 25.8)	Probably Damaging	Damaging	Disease Causing
c.3809A > G; p.Asn1270Ser	Deleterious (Score: 0.896)	Deleterious (Score: 23.3)	Probably Damaging	Damaging	Disease Causing
c.3207C > A; p.His1069Gln	Deleterious (Score: 0.909)	Deleterious (Score: 23.0)	Probably Damaging	Damaging	Disease Causing
c.2071G > A; p.Gly691Arg	Deleterious (Score: 0.977)	Deleterious (Score: 29.4)	Probably Damaging	Damaging	Disease Causing
c.3704G > A; p.Gly1235Asp	Deleterious (Score: 0.949)	Deleterious (Score: 26.6)	Probably Damaging	Damaging	Disease Causing
c.3182G > A; p.Gly1061Glu	Deleterious (Score: 0.928)	Deleterious (Score: 28.3)	Probably Damaging	Damaging	Disease Causing
c.2332C > G; p.Arg778Gly	Deleterious (Score: 0.872)	Deleterious (Score: 24.6)	Probably Damaging	Damaging	Disease Causing
c.2755C > T; p.Arg919Trp	Deleterious (Score: 0.75)	Deleterious (Score: 32.0)	Probably Damaging	Damaging	Disease Causing
c.3266G > A; p.Gly1089Glu	Deleterious (Score: 0.933)	Deleterious (Score: 28.0)	Probably Damaging	Tolerated	Disease Causing
c.3784G > T; p.Val1262Phe	Deleterious (Score: 0.975)	Deleterious (Score: 24.4)	Probably Damaging	Damaging	Disease Causing

**Table 3:** Exon locations, allele frequencies and the resulting functional consequences of the detected genetic variations of *ATP7B* according to gnomAD v4.1 and HGMD. Allele frequencies were obtained from gnomAD v4.1 with ‘NO’ indicating ‘not observed in gnomAD v4.1’. Additionally, in the right-hand column of the table, references to publications are provided that report the specific *ATP7B* variants.

Phenotype	Case ID	Gene Variant		Exon Location	Allele Frequency (%)	Functional Consequence According to		Reported in
		Nucleotide Change	Protein Change			gnomAD v4.1	HGMD	
**WD with Hepatic Symptoms**	H1	c.3979C > G	p.Leu1327Val	19	NO	Loss of Function	Disease Mutation	[30,31]
	H2	c.2332C > T	p.Arg778Trp	19	0.007125	Loss of Function	Disease Mutation	[31,32,33,34,35,36,37,38]
	H3	c.1846C > T	p.Arg616Trp	8	0.001673	Compound Heterozygous Loss of Function	Disease Mutation	[38,39,40,41,42,43]
		c.3809A > G	p.Asn1270Ser	18	0.01431	Loss of Function	Disease Mutation	[31,32,36,38,41,42,44,45,46,47,48,49,50,51,52,53,54,55,56,57,58,59,60,61,62,107]
	H4	c.3207C > A	p.His1069Gln	11	0.1019	Loss of Function	Disease Mutation	[31,32,34,36,41,42,46,50,54,59,63,64,65,66,67,68,69,70,71,72,73,74,75,76,77,78,79,80,81,82,83,84,85,86,87,88,89,90,91,92,93,94,95,96,97]
	H5	c.1369C > T	p.Gln457Ter	5	NO	Compound Heterozygous Loss of Function	Disease Mutation	[50,98,99]
		c.3704G > A	p.Gly1235Asp	12	NO	Loss of Function	Disease Mutation	[42,100]
	H6	c.2071G > A	p.Gly691Arg	8	NO	Loss of Function	Disease Mutation	[31,32,50,101,102,103]
	H7	c.2071G > A	p.Gly691Arg	8	NO	Loss of Function	Disease Mutation	[31,32,50,101,102,103]
**WD with Neurological Symptoms**	N1	c.3207C > A	p.His1069Gln	11	0.1019	Loss of Function	Disease Mutation	[31,32,34,36,41,42,46,50,54,59,63,64,65,66,67,68,69,70,71,72,73,74,75,76,77,78,79,80,81,82,83,84,85,86,87,88,89,90,91,92,93,94,95,96,97]
	N2	c.3182G > A	p.Gly1061Glu	14	NO	Loss of Function	Disease Mutation	[31,32,52,98,104,105]
	N3	c.2332C > T	p.Arg778Trp	19	0.007125	Loss of Function	Disease Mutation	[31,32,33,34,35,36,37,38]
	N4	c.3207C > A	p.His1069Gln	11	0.1019	Loss of Function	Disease Mutation	[31,32,34,36,41,42,46,50,54,59,63,64,65,66,67,68,69,70,71,72,73,74,75,76,77,78,79,80,81,82,83,84,85,86,87,88,89,90,91,92,93,94,95,96,97]
	N5	c.2332C > G	p.Arg778Gly	19	0.001177	Loss of Function	Disease Mutation	[31,34,36,42,46,50,73,95,106,107]
	N6	c.2755C > T	p.Arg919Trp	12	0.003222	Loss of Function	Disease Mutation	[31,38,48,77,103]
	N7	c.2755C > T	p.Arg919Trp	12	0.003222	Loss of Function	Disease Mutation	[31,38,48,77,103]
	N8	c.2304dupC	p.Met769HisfsTer26	8	0.009789	Loss of Function	Disease Mutation	[32,33,38,42,43,46,48,49,50,53,57,65,77,89,108,109,110,111,112,113,114,115,116,117]
	N9	c.3266G > A	p.Gly1089Glu	13	NO	Compound Heterozygous Loss of Function	Disease Mutation	[30,42,93]
		c.3784G > T	p.Val1262Phe	13	0.0005576	Compound Heterozygous Loss of Function	Disease Mutation	[30,36,93]
	N10	c.3266G > A	p.Gly1089Glu	13	NO	Compound Heterozygous Loss of Function	Disease Mutation	[30,42,93]
		c.3784G > T	p.Val1262Phe	13	0.0005576	Compound Heterozygous Loss of Function	Disease Mutation	[30,36,93]

**Table 4:** Biochemical and demographic data of individual WD cases.

Case ID	Age (Year)	Gender	AST	ALT	ALP	GGT	TBil	DBil	CRE	PLT	CP	24 h U-Cu	PC	RelWD	KFR
H1	13	M	7	17	78	9	0.46	0.12	0.5	175	0.08	323	Cousins	–	NA
H2	16	M	64	55	173	97	1.04	0.21	0.8	345	NA	343	–	–	Pos
H3	16	F	33	33	200	47	0.4	0.09	0.6	150	0.12	438	–	2 Siblings, 3 Maternal Relatives	Neg
H4	11	M	93	102	198	175	0.43	0.09	0.9	202	11.8	403	Cousins	Paternal Uncle	Pos
H5	13	M	54	62	863	43	0.48	0.1	0.7	283	32.02	992	Cousins	–	Neg
H6	9	F	19	15	155	10	0.9	0.19	0.6	265	NA	NA	Cousins	Sibling	Neg
H7	8	F	32	25	277	19	0.6	0.09	0.5	248	0.33	402	Cousins	Sibling	Neg
N1	24	M	58	69	238	349	0.86	0.27	0.8	203	NA	1066	Cousins	Sibling	Pos
N2	15	F	492	457	96	83	1.2	0.46	1.6	153	0.08	137	–	–	Pos
N3	6	M	33	27	339	17	0.6	0.1	0.6	312	0.28	410	NA	–	Pos
N4	23	F	21	10	49	10	0.34	0.08	0.9	262	15.7	405	Cousins	–	Pos
N5	20	F	99	104	351	280	6.7	3.54	0.7	23	10	25	Cousins	–	Pos
N6	24	F	22	23	100	7	0.4	0.1	0.6	194	0.07	190	Cousins	–	Pos
N7	20	F	36	41	125	8	1.7	0.27	0.6	321	0.06	29	Cousins	Sibling	Neg
N8	10	M	25	24	500	24	1.9	0.39	0.7	31	0.08	3062	Cousins	–	Neg
N9	18	M	20	26	136	86	0.46	0.12	0.8	250	0.09	2467	Cousins	Sibling	Neg
N10	21	M	22	22	95	20	1.86	0.35	0.8	259	NA	1152	Cousins	Sibling	Pos

AST: Aspartate Aminotransferase, ALT: Alanine Aminotransferase, ALP: Alkaline Phosphatase, GGT: Gamma-Glutamyltransferase, TBil: Total Bilirubin, Dbil: Direct Bilirubin, CRE: Creatinine, PLT: Platelets, CP: Ceruloplasmin, 24 h U-Cu: 24 h Urine Copper, PC: Parental Consanguinity, RelWD: Relative with WD, KFR: Kayser-Fleischer Ring, NA: Not available, Neg: Negative, –: None, Pos: Positive.

## Data Availability

The datasets analyzed during the current study are available from the corresponding author on reasonable request.

## References

[1] Mulligan C., Bronstein J.M. (2020). Wilson Disease: An Overview and Approach to Management. Neurol. Clin..

[2] Sánchez-Monteagudo A., Ripollés E., Berenguer M., Espinós C. (2021). Wilson’s Disease: Facing the Challenge of Diagnosing a Rare Disease. Biomedicines.

[3] Chang I.J., Hahn S.H., Członkowska A., Schilsky M.L. (2017). Chapter 3—The genetics of Wilson disease. Handbook of Clinical Neurology.

[4] Członkowska A., Litwin T., Dusek P., Ferenci P., Lutsenko S., Medici V., Rybakowski J.K., Weiss K.H., Schilsky M.L. (2018). Wilson disease. Nat. Rev. Dis. Primers.

[5] Lucena-Valera A., Ruz-Zafra P., Ampuero J. (2023). Wilson’s disease: Overview. Med. Clin..

[6] Shribman S., Poujois A., Bandmann O., Czlonkowska A., Warner T.T. (2021). Wilson’s disease: Update on pathogenesis, biomarkers and treatments. J. Neurol. Neurosurg. Psychiatry.

[7] Bitter R.M., Oh S., Deng Z., Rahman S., Hite R.K., Yuan P. (2022). Structure of the Wilson disease copper transporter ATP7B. Sci. Adv..

[8] Gale J., Aizenman E. (2024). The physiological and pathophysiological roles of copper in the nervous system. Eur. J. Neurosci..

[9] Fang S., Strader C., Costantino H., Weiss K.H., Hedera P. (2024). Wilson disease in the USA: Epidemiology and real-world patient characteristics based on a retrospective observational health claims study. BMJ Open.

[10] Hedera P. (2019). Wilson’s disease: A master of disguise. Park. Relat. Disord..

[11] Abdel Ghaffar T.Y., Elsayed S.M., Elnaghy S., Shadeed A., Elsobky E.S., Schmidt H. (2011). Phenotypic and genetic characterization of a cohort of pediatric Wilson disease patients. BMC Pediatr..

[12] Ovchinnikova E.V., Garbuz M.M., Ovchinnikova A.A., Kumeiko V.V. (2024). Epidemiology of Wilson’s Disease and Pathogenic Variants of the ATP7B Gene Leading to Diversified Protein Disfunctions. Int. J. Mol. Sci..

[13] Zhan T., Guan Y., Sun C., Wang L., Wang Y., Li X. (2024). Assessment and factors affecting quality of life among patients with Wilson’s disease. Sci. Rep..

[14] Jopowicz A., Tarnacka B. (2023). Neurological Wilson’s Disease Signs-Hepatic Encephalopathy or Copper Toxicosis?. Diagnostics.

[15] Delle Cave V., Di Dato F., Iorio R. (2024). Wilson’s Disease with Acute Hepatic Onset: How to Diagnose and Treat It. Children.

[16] European Association for the Study of the Liver (2025). EASL-ERN Clinical Practice Guidelines on Wilson’s disease. J. Hepatol..

[17] Schroeder S.M., Matsukuma K.E., Medici V. (2021). Wilson disease and the differential diagnosis of its hepatic manifestations: A narrative review of clinical, laboratory, and liver histological features. Ann. Transl. Med..

[18] Medici V., LaSalle J.M. (2019). Genetics and epigenetic factors of Wilson disease. Ann. Transl. Med..

[19] Gromadzka G., Bendykowska M., Przybyłkowski A. (2023). Wilson’s Disease-Genetic Puzzles with Diagnostic Implications. Diagnostics.

[20] Ferenci P., Członkowska A., Schilsky M.L. (2017). Chapter 14—Diagnosis of Wilson disease. Handbook of Clinical Neurology.

[21] Salman H.M., Amin M., Syed J., Sarfraz Z., Sarfraz A., Sarfraz M., Farfán Bajaña M.J., Felix M., Cherrez-Ojeda I. (2022). Biochemical testing for the diagnosis of Wilson’s disease: A systematic review. J. Clin. Lab. Anal..

[22] Wungjiranirun M., Sharzehi K. (2023). Wilson’s Disease. Semin. Neurol..

[23] Ferenci P., Caca K., Loudianos G., Mieli-Vergani G., Tanner S., Sternlieb I., Schilsky M., Cox D., Berr F. (2003). Diagnosis and phenotypic classification of Wilson disease. Liver Int..

[24] QIAGEN Digital Insights QIAGEN Bioinformatics Software. https://digitalinsights.qiagen.com/.

[25] Ioannidis N.M., Rothstein J.H., Pejaver V., Middha S., McDonnell S.K., Baheti S., Musolf A., Li Q., Holzinger E., Karyadi D. (2016). REVEL: An Ensemble Method for Predicting the Pathogenicity of Rare Missense Variants. Am. J. Hum. Genet..

[26] Kircher M., Witten D.M., Jain P., O’Roak B.J., Cooper G.M., Shendure J. (2014). A general framework for estimating the relative pathogenicity of human genetic variants. Nat. Genet..

[27] Adzhubei I.A., Schmidt S., Peshkin L., Ramensky V.E., Gerasimova A., Bork P., Kondrashov A.S., Sunyaev S.R. (2010). A method and server for predicting damaging missense mutations. Nat. Methods.

[28] Ng P.C., Henikoff S. (2003). SIFT: Predicting amino acid changes that affect protein function. Nucleic Acids Res..

[29] Schwarz J.M., Cooper D.N., Schuelke M., Seelow D. (2014). MutationTaster2: Mutation prediction for the deep-sequencing age. Nat. Methods.

[30] Loudianos G., Dessi V., Lovicu M., Angius A., Altuntas B., Giacchino R., Marazzi M., Marcellini M., Sartorelli M.R., Sturniolo G.C. (1999). Mutation analysis in patients of Mediterranean descent with Wilson disease: Identification of 19 novel mutations. J. Med. Genet..

[31] Schushan M., Bhattacharjee A., Ben-Tal N., Lutsenko S. (2012). A structural model of the copper ATPase ATP7B to facilitate analysis of Wilson disease-causing mutations and studies of the transport mechanism. Metallomics.

[32] Capalbo A., Valero R.A., Jimenez-Almazan J., Pardo P.M., Fabiani M., Jiménez D., Simon C., Rodriguez J.M. (2019). Optimizing clinical exome design and parallel gene-testing for recessive genetic conditions in preconception carrier screening: Translational research genomic data from 14,125 exomes. PLoS Genet..

[33] García-Villarreal L., Hernández-Ortega A., Sánchez-Monteagudo A., Peña-Quintana L., Ramírez-Lorenzo T., Riaño M., Moreno-Pérez R., Monescillo A., González-Santana D., Quiñones I. (2021). Wilson disease: Revision of diagnostic criteria in a clinical series with great genetic homogeneity. J. Gastroenterol..

[34] Sánchez-Monteagudo A., Álvarez-Sauco M., Sastre I., Martínez-Torres I., Lupo V., Berenguer M., Espinós C. (2020). Genetics of Wilson disease and Wilson-like phenotype in a clinical series from eastern Spain. Clin. Genet..

[35] Shah A.B., Chernov I., Zhang H.T., Ross B.M., Das K., Lutsenko S., Parano E., Pavone L., Evgrafov O., Ivanova-Smolenskaya I.A. (1997). Identification and analysis of mutations in the Wilson disease gene (ATP7B): Population frequencies, genotype-phenotype correlation, and functional analyses. Am. J. Hum. Genet..

[36] Squitti R., Siotto M., Bucossi S., Polimanti R. (2014). In silico investigation of the ATP7B gene: Insights from functional prediction of non-synonymous substitution to protein structure. Biometals.

[37] Stranneheim H., Lagerstedt-Robinson K., Magnusson M., Kvarnung M., Nilsson D., Lesko N., Engvall M., Anderlid B.M., Arnell H., Johansson C.B. (2021). Integration of whole genome sequencing into a healthcare setting: High diagnostic rates across multiple clinical entities in 3219 rare disease patients. Genome Med..

[38] Zhao S., Xiang J., Fan C., Asan, Shang X., Zhang X., Chen Y., Zhu B., Cai W., Chen S. (2019). Pilot study of expanded carrier screening for 11 recessive diseases in China: Results from 10,476 ethnically diverse couples. Eur. J. Hum. Genet..

[39] Caca K., Ferenci P., Kühn H.J., Polli C., Willgerodt H., Kunath B., Hermann W., Mössner J., Berr F. (2001). High prevalence of the H1069Q mutation in East German patients with Wilson disease: Rapid detection of mutations by limited sequencing and phenotype-genotype analysis. J. Hepatol..

[40] de Bie P., van de Sluis B., Burstein E., van de Berghe P.V., Muller P., Berger R., Gitlin J.D., Wijmenga C., Klomp L.W. (2007). Distinct Wilson’s disease mutations in ATP7B are associated with enhanced binding to COMMD1 and reduced stability of ATP7B. Gastroenterology.

[41] Huster D., Kühne A., Bhattacharjee A., Raines L., Jantsch V., Noe J., Schirrmeister W., Sommerer I., Sabri O., Berr F. (2012). Diverse functional properties of Wilson disease ATP7B variants. Gastroenterology.

[42] Kars M.E., Başak A.N., Onat O.E., Bilguvar K., Choi J., Itan Y., Çağlar C., Palvadeau R., Casanova J.L., Cooper D.N. (2021). The genetic structure of the Turkish population reveals high levels of variation and admixture. Proc. Natl. Acad. Sci. USA.

[43] Wang F., Li Y., Zhao S., Chen Z., Xu Z., Wang L., Zhang T.J., Yan J., Cao L., Wang P. (2022). The utility of hierarchical genetic testing in paediatric liver disease. Liver Int..

[44] Abuduxikuer K., Li L.T., Qiu Y.L., Wang N.L., Wang J.S. (2015). Wilson disease with hepatic presentation in an eight-month-old boy. World J. Gastroenterol..

[45] Amendola L.M., Dorschner M.O., Robertson P.D., Salama J.S., Hart R., Shirts B.H., Murray M.L., Tokita M.J., Gallego C.J., Kim D.S. (2015). Actionable exomic incidental findings in 6503 participants: Challenges of variant classification. Genome Res..

[46] Balashova M.S., Tuluzanovskaya I.G., Glotov O.S., Glotov A.S., Barbitoff Y.A., Fedyakov M.A., Alaverdian D.A., Ivashchenko T.E., Romanova O.V., Sarana A.M. (2020). The spectrum of pathogenic variants of the ATP7B gene in Wilson disease in the Russian Federation. J. Trace Elem. Med. Biol..

[47] Barada K., El-Atrache M., El H., Rida K., El-Hajjar J., Mahfoud Z., Usta J. (2010). Homozygous mutations in the conserved ATP hinge region of the Wilson disease gene: Association with liver disease. J. Clin. Gastroenterol..

[48] Chau J.F.T., Yu M.H.C., Chui M.M.C., Yeung C.C.W., Kwok A.W.C., Zhuang X., Lee R., Fung J.L.F., Lee M., Mak C.C.Y. (2022). Comprehensive analysis of recessive carrier status using exome and genome sequencing data in 1543 Southern Chinese. npj Genom. Med..

[49] Chen Y.C., Yu H., Wang R.M., Xie J.J., Ni W., Zhang Y., Dong Y., Wu Z.Y. (2019). Contribution of intragenic deletions to mutation spectrum in Chinese patients with Wilson’s disease and possible mechanism underlying ATP7B gross deletions. Park. Relat. Disord..

[50] Couchonnal E., Bouchard S., Sandahl T.D., Pagan C., Lion-François L., Guillaud O., Habes D., Debray D., Lamireau T., Broué P. (2021). ATP7B variant spectrum in a French pediatric Wilson disease cohort. Eur. J. Med. Genet..

[51] Daneshjoo O., Garshasbi M. (2018). Novel compound heterozygote mutations in the ATP7B gene in an Iranian family with Wilson disease: A case report. J. Med. Case Rep..

[52] Guggilla S.R., Senagari J.R., Rao P.N., Madireddi S. (2015). Spectrum of mutations in the ATP binding domain of ATP7B gene of Wilson Disease in a regional Indian cohort. Gene.

[53] Hua R., Hua F., Jiao Y., Pan Y., Yang X., Peng S., Niu J. (2016). Mutational analysis of ATP7B in Chinese Wilson disease patients. Am. J. Transl. Res..

[54] Iida M., Terada K., Sambongi Y., Wakabayashi T., Miura N., Koyama K., Futai M., Sugiyama T. (1998). Analysis of functional domains of Wilson disease protein (ATP7B) in Saccharomyces cerevisiae. FEBS Lett..

[55] Liu P., Che F., Shu C., Li Y., Liu X. (2022). Analysis of clinical phenotypes and ATP7B gene variants in 75 children patients with Wilson’s disease. Zhonghua Yi Xue Yi Chuan Xue Za Zhi.

[56] Penon-Portmann M., Lotz-Esquivel S., Chavez Carrera A., Jiménez-Hernández M., Alvarado-Romero D., Segura-Cordero S., Rimolo-Donadio F., Hevia-Urrutia F., Mora-Guevara A., Saborío-Rocafort M. (2020). Wilson disease in Costa Rica: Pediatric phenotype and genotype characterization. JIMD Rep..

[57] Qiao L., Ge J., Li C., Liu Y., Hu C., Hu S., Li W., Li T. (2021). Pathogenic gene variation spectrum and carrier screening for Wilson’s disease in Qingdao area. Mol. Genet. Genom. Med..

[58] Rao R., Shu S., Han Y.Z., Chiu Y.J., Han Y.S. (2018). A case report: Co-occurrence of Wilson disease and oculocutaneous albinism in a Chinese patient. Medicine.

[59] Tanzi R.E., Petrukhin K., Chernov I., Pellequer J.L., Wasco W., Ross B., Romano D.M., Parano E., Pavone L., Brzustowicz L.M. (1993). The Wilson disease gene is a copper transporting ATPase with homology to the Menkes disease gene. Nat. Genet..

[60] Wu Z., Wang N., Murong S., Lin M. (1999). Missense mutations of exons 14 and 18 of Wilson’s disease gene in Chinese patients. Zhonghua Yi Xue Yi Chuan Xue Za Zhi.

[61] Xiao Z., Yang Y., Huang H., Tang H., Liu L., Tang J., Shi X. (2021). Molecular analysis of 53 Chinese families with Wilson’s disease: Six novel mutations identified. Mol. Genet. Genom. Med..

[62] Zhou D., Jia S., Yi L., Wu Z., Song Y., Zhang B., Li Y., Yang X., Xu A., Li X. (2022). Identification of potential modifier genes in Chinese patients with Wilson disease. Metallomics.

[63] Battisti C., Loudianos G., Rufa A., Dotti M.T., Sangiorgi S., Dessì V., Lovicu M., Pirastu M., Federico A. (1999). Detection of a rare Wilson disease mutation associated with arylsulfatase A pseudodeficiency. Am. J. Med. Genet..

[64] Ceyhan-Birsoy O., Murry J.B., Machini K., Lebo M.S., Yu T.W., Fayer S., Genetti C.A., Schwartz T.S., Agrawal P.B., Parad R.B. (2019). Interpretation of Genomic Sequencing Results in Healthy and Ill Newborns: Results from the BabySeq Project. Am. J. Hum. Genet..

[65] Cocoş R., Şendroiu A., Schipor S., Bohîlţea L.C., Şendroiu I., Raicu F. (2014). Genotype-phenotype correlations in a mountain population community with high prevalence of Wilson’s disease: Genetic and clinical homogeneity. PLoS ONE.

[66] Członkowska A., Gromadzka G., Chabik G. (2009). Monozygotic female twins discordant for phenotype of Wilson’s disease. Mov. Disord..

[67] Członkowska A., Rodo M., Gromadzka G. (2008). Late onset Wilson’s disease: Therapeutic implications. Mov. Disord..

[68] Das S., Mohammed A., Mandal T., Maji S., Verma J., Ruturaj, Gupta A. (2022). Polarized trafficking and copper transport activity of ATP7B: A mutational approach to establish genotype-phenotype correlation in Wilson disease. Hum. Mutat..

[69] Despotov K., Klivényi P., Nagy I., Pálvölgyi A., Vécsei L., Rajda C. (2022). Rare co-occurrence of multiple sclerosis and Wilson’s disease—Case report. BMC Neurol..

[70] Diaz J., Fonseca A.G., Arboleda R., Frade A., Gennaro M.P., Jayakar P., Schleifer P., Hernandez E. (2021). Case Report: The Association of Wilson Disease in a Patient with Ataxia and GLUT-1 Deficiency. Front. Pediatr..

[71] Dmitriev O.Y., Bhattacharjee A., Nokhrin S., Uhlemann E.M., Lutsenko S. (2011). Difference in stability of the N-domain underlies distinct intracellular properties of the E1064A and H1069Q mutants of copper-transporting ATPase ATP7B. J. Biol. Chem..

[72] Duc H.H., Hefter H., Stremmel W., Castañeda-Guillot C., Hernández Hernández A., Cox D.W., Auburger G. (1998). His1069Gln and six novel Wilson disease mutations: Analysis of relevance for early diagnosis and phenotype. Eur. J. Hum. Genet..

[73] Dzinovic I., Boesch S., Škorvánek M., Necpál J., Švantnerová J., Pavelekova P., Havránková P., Tsoma E., Indelicato E., Runkel E. (2022). Genetic overlap between dystonia and other neurologic disorders: A study of 1,100 exomes. Park. Relat. Disord..

[74] Firneisz G., Lakatos P.L., Szalay F., Polli C., Glant T.T., Ferenci P. (2002). Common mutations of ATP7B in Wilson disease patients from Hungary. Am. J. Med. Genet..

[75] Freudenberg-Hua Y., Freudenberg J., Vacic V., Abhyankar A., Emde A.K., Ben-Avraham D., Barzilai N., Oschwald D., Christen E., Koppel J. (2014). Disease variants in genomes of 44 centenarians. Mol. Genet. Genom. Med..

[76] Gucev Z.S., Pop-Jordanova N., Calovska V., Tasic V., Slavevska N., Laban N., Noli M.C., Lepori M.B., Loudianos G. (2011). Acute Gallbladder Hydrops and Arthritis: Unusual initial manifestations of Wilson’s Disease (WD): Case Report. Prilozi.

[77] Hou Y.C., Yu H.C., Martin R., Cirulli E.T., Schenker-Ahmed N.M., Hicks M., Cohen I.V., Jönsson T.J., Heister R., Napier L. (2020). Precision medicine integrating whole-genome sequencing, comprehensive metabolomics, and advanced imaging. Proc. Natl. Acad. Sci. USA.

[78] Litwin T., Gromadzka G., Członkowska A. (2012). Apolipoprotein E gene (APOE) genotype in Wilson’s disease: Impact on clinical presentation. Park. Relat. Disord..

[79] Maier-Dobersberger T., Ferenci P., Polli C., Balać P., Dienes H.P., Kaserer K., Datz C., Vogel W., Gangl A. (1997). Detection of the His1069Gln mutation in Wilson disease by rapid polymerase chain reaction. Ann. Intern. Med..

[80] Marazzi M.G., Giardino S., Dufour C., Serafino M., Sperlì D., Giacchino R. (2012). Good response with zinc acetate monotherapy in an adolescent affected by severe Wilson disease. Pediatr. Med. Chir..

[81] McCreary D., Omoyinmi E., Hong Y., Mulhern C., Papadopoulou C., Casimir M., Hacohen Y., Nyanhete R., Ahlfors H., Cullup T. (2019). Development and Validation of a Targeted Next-Generation Sequencing Gene Panel for Children with Neuroinflammation. JAMA Netw. Open.

[82] Mercer S.W., Wang J., Burke R. (2017). In Vivo Modeling of the Pathogenic Effect of Copper Transporter Mutations That Cause Menkes and Wilson Diseases, Motor Neuropathy, and Susceptibility to Alzheimer’s Disease. J. Biol. Chem..

[83] Morgan C.T., Tsivkovskii R., Kosinsky Y.A., Efremov R.G., Lutsenko S. (2004). The distinct functional properties of the nucleotide-binding domain of ATP7B, the human copper-transporting ATPase: Analysis of the Wilson disease mutations E1064A, H1069Q, R1151H, and C1104F. J. Biol. Chem..

[84] Panzer M., Viveiros A., Schaefer B., Baumgartner N., Seppi K., Djamshidian A., Todorov T., Griffiths W.J.H., Schott E., Schuelke M. (2022). Synonymous mutation in adenosine triphosphatase copper-transporting beta causes enhanced exon skipping in Wilson disease. Hepatol. Commun..

[85] Parisi S., Polishchuk E.V., Allocca S., Ciano M., Musto A., Gallo M., Perone L., Ranucci G., Iorio R., Polishchuk R.S. (2018). Characterization of the most frequent ATP7B mutation causing Wilson disease in hepatocytes from patient induced pluripotent stem cells. Sci. Rep..

[86] Petters J., Völkner C., Krohn S., Murua Escobar H., Bullerdiek J., Reuner U., Frech M.J., Hermann A., Lukas J. (2020). Generation of two induced pluripotent stem cell lines from a female adult homozygous for the Wilson disease associated ATP7B variant p.H1069Q (AKOSi008-A) and a healthy control (AKOSi009-A). Stem Cell Res..

[87] Polishchuk E.V., Concilli M., Iacobacci S., Chesi G., Pastore N., Piccolo P., Paladino S., Baldantoni D., van I.S.C., Chan J. (2014). Wilson disease protein ATP7B utilizes lysosomal exocytosis to maintain copper homeostasis. Dev. Cell.

[88] Rodriguez-Granillo A., Sedlak E., Wittung-Stafshede P. (2008). Stability and ATP binding of the nucleotide-binding domain of the Wilson disease protein: Effect of the common H1069Q mutation. J. Mol. Biol..

[89] Samadzadeh S., Kruschel T., Novak M., Kallenbach M., Hefter H. (2022). Different Response Behavior to Therapeutic Approaches in Homozygotic Wilson’s Disease Twins with Clinical Phenotypic Variability: Case Report and Literature Review. Genes.

[90] Sapuppo A., Pavone P., Praticò A.D., Ruggieri M., Bertino G., Fiumara A. (2020). Genotype-phenotype variable correlation in Wilson disease: Clinical history of two sisters with the similar genotype. BMC Med. Genet..

[91] Skowronska M., Litwin T., Kurkowska-Jastrzębska I., Członkowska A. (2020). Transcranial sonography changes in heterozygotic carriers of the ATP7B gene. Neurol. Sci..

[92] Usta J., Abu Daya H., Halawi H., Al-Shareef I., El-Rifai O., Malli A.H., Sharara A.I., Habib R.H., Barada K. (2012). Homozygosity for Non-H1069Q Missense Mutations in ATP7B Gene and Early Severe Liver Disease: Report of Two Families and a Meta-analysis. JIMD Rep..

[93] van den Berghe P.V., Stapelbroek J.M., Krieger E., de Bie P., van de Graaf S.F., de Groot R.E., van Beurden E., Spijker E., Houwen R.H., Berger R. (2009). Reduced expression of ATP7B affected by Wilson disease-causing mutations is rescued by pharmacological folding chaperones 4-phenylbutyrate and curcumin. Hepatology.

[94] Walshe J.M. (2016). Wilson disease: A most unusual patient. QJM Int. J. Med..

[95] Zech M., Jech R., Boesch S., Škorvánek M., Weber S., Wagner M., Zhao C., Jochim A., Necpál J., Dincer Y. (2020). Monogenic variants in dystonia: An exome-wide sequencing study. Lancet Neurol..

[96] Zhao C., Chai H., Zhou Q., Wen J., Reddy U.M., Kastury R., Jiang Y., Mak W., Bale A.E., Zhang H. (2021). Exome sequencing analysis on products of conception: A cohort study to evaluate clinical utility and genetic etiology for pregnancy loss. Genet. Med..

[97] Belousova O.B., Okishev D.N., Ignatova T.M., Balashova M.S., Boulygina E.S. (2017). Hereditary Multiple Cerebral Cavernous Malformations Associated with Wilson Disease and Multiple Lipomatosis. World Neurosurg..

[98] Curtis D., Durkie M., Balac P., Sheard D., Goodeve A., Peake I., Quarrell O., Tanner S. (1999). A study of Wilson disease mutations in Britain. Hum. Mutat..

[99] Xiong H.Y., Alipanahi B., Lee L.J., Bretschneider H., Merico D., Yuen R.K., Hua Y., Gueroussov S., Najafabadi H.S., Hughes T.R. (2015). RNA splicing. The human splicing code reveals new insights into the genetic determinants of disease. Science.

[100] Rodríguez-Quiroga S.A., Rosales J., Arakaki T., Cordoba M., González-Morón D., Medina N., Garretto N.S., Kauffman M.A. (2015). Timely diagnosis of Wilson’s disease using whole exome sequencing. Park. Relat. Disord..

[101] Barada K., Nemer G., ElHajj, Touma J., Cortas N., Boustany R.M., Usta J. (2007). Early and severe liver disease associated with homozygosity for an exon 7 mutation, G691R, in Wilson’s disease. Clin. Genet..

[102] Denoyer Y., Woimant F., Bost M., Edan G., Drapier S. (2013). Neurological Wilson’s disease lethal for the son, asymptomatic in the father. Mov. Disord..

[103] Loudianos G., Dessì V., Lovicu M., Angius A., Nurchi A., Sturniolo G.C., Marcellini M., Zancan L., Bragetti P., Akar N. (1998). Further delineation of the molecular pathology of Wilson disease in the Mediterranean population. Hum. Mutat..

[104] Cheema H., Bertoli-Avella A.M., Skrahina V., Anjum M.N., Waheed N., Saeed A., Beetz C., Perez-Lopez J., Rocha M.E., Alawbathani S. (2020). Genomic testing in 1019 individuals from 349 Pakistani families results in high diagnostic yield and clinical utility. npj Genom. Med..

[105] Roy S., McCann C.J., Ralle M., Ray K., Ray J., Lutsenko S., Jayakanthan S. (2020). Analysis of Wilson disease mutations revealed that interactions between different ATP7B mutants modify their properties. Sci. Rep..

[106] Figus A., Angius A., Loudianos G., Bertini C., Dessi V., Loi A., Deiana M., Lovicu M., Olla N., Sole G. (1995). Molecular pathology and haplotype analysis of Wilson disease in Mediterranean populations. Am. J. Hum. Genet..

[107] Garcia E., Aguilar-Cevallos J., Silva-Garcia R., Ibarra A. (2016). Cytokine and Growth Factor Activation In Vivo and In Vitro after Spinal Cord Injury. Mediat. Inflamm..

[108] Chen H.L., Li H.Y., Wu J.F., Wu S.H., Chen H.L., Yang Y.H., Hsu Y.H., Liou B.Y., Chang M.H., Ni Y.H. (2019). Panel-Based Next-Generation Sequencing for the Diagnosis of Cholestatic Genetic Liver Diseases: Clinical Utility and Challenges. J. Pediatr..

[109] Dong X., Liu B., Yang L., Wang H., Wu B., Liu R., Chen H., Chen X., Yu S., Chen B. (2020). Clinical exome sequencing as the first-tier test for diagnosing developmental disorders covering both CNV and SNV: A Chinese cohort. J. Med. Genet..

[110] Fang Y., Yu J., Lou J., Peng K., Zhao H., Chen J. (2021). Clinical and Genetic Spectra of Inherited Liver Disease in Children in China. Front. Pediatr..

[111] Haer-Wigman L., van der Schoot V., Feenstra I., Vulto-van Silfhout A.T., Gilissen C., Brunner H.G., Vissers L., Yntema H.G. (2019). 1 in 38 individuals at risk of a dominant medically actionable disease. Eur. J. Hum. Genet..

[112] Hildebrand M.S., Jackson V.E., Scerri T.S., Van Reyk O., Coleman M., Braden R.O., Turner S., Rigbye K.A., Boys A., Barton S. (2020). Severe childhood speech disorder: Gene discovery highlights transcriptional dysregulation. Neurology.

[113] Huang C., Fang M., Xiao X., Gao Z., Wang Y., Gao C. (2022). Genetic studies discover novel coding and non-coding mutations in patients with Wilson’s disease in China. J. Clin. Lab. Anal..

[114] Thomas G.R., Forbes J.R., Roberts E.A., Walshe J.M., Cox D.W. (1995). The Wilson disease gene: Spectrum of mutations and their consequences. Nat. Genet..

[115] Tsuchiya M., Takaki R., Kobayashi F., Nagasaka T., Shindo K., Takiyama Y. (2017). Multiple pseudofractures due to Fanconi’s syndrome associated with Wilson’s disease. Rinsho Shinkeigaku.

[116] Usta J., Wehbeh A., Rida K., El-Rifai O., Estiphan T.A., Majarian T., Barada K. (2014). Phenotype-genotype correlation in Wilson disease in a large Lebanese family: Association of c.2299insC with hepatic and of p. Ala1003Thr with neurologic phenotype. PLoS ONE.

[117] Yu H., Xie J.J., Chen Y.C., Dong Q.Y., Dong Y., Ni W., Wu Z.Y. (2017). Clinical features and outcome in patients with osseomuscular type of Wilson’s disease. BMC Neurol..

[118] Loudianos G., Dessì V., Angius A., Lovicu M., Loi A., Deiana M., Akar N., Vajro P., Figus A., Cao A. (1996). Wilson disease mutations associated with uncommon haplotypes in Mediterranean patients. Hum. Genet..

[119] Margarit E., Bach V., Gómez D., Bruguera M., Jara P., Queralt R., Ballesta F. (2005). Mutation analysis of Wilson disease in the Spanish population—Identification of a prevalent substitution and eight novel mutations in the ATP7B gene. Clin. Genet..

[120] Al-Obaidi R.G., Al-Musawi B.M. (2025). Spectrum and classification of ATP7B variants with clinical correlation in children with Wilson disease. Saudi Med. J..

[121] Chindamo M.C., Judice C.C., Valladares M.A.B., da Rocha B.P.R., do Vale E.A., Pereira A.M.F., Santos U.C., Evangelista A.S., Calçado F.L.V., Rotman V. (2024). Frequency of ATP7B Gene Mutations in a Brazilian Cohort of Patients with Wilson’s Disease. Ann. Hepatol..

[122] Saha A., Das S., De S., Dutta T., Roy S., Biswas A., Sengupta M. (2024). An Effort to Identify Genetic Determinants in Siblings with Wilson Disease Manifesting Striking Clinical Heterogeneity: An Exome Profiling Study of Two Indian Families. Pediatr. Neurol..

[123] Bakir A., Topcu V., Cavdarli B. (2024). Wilson Disease in a Turkish Population: Molecular Insights into an Old Disease with Reported and Novel Variants. Gazi Med. J..

[124] Simsek Papur O., Akman S.A., Cakmur R., Terzioglu O. (2013). Mutation analysis of ATP7B gene in Turkish Wilson disease patients: Identification of five novel mutations. Eur. J. Med. Genet..

[125] Simsek Papur Ö., Asik Akman S., Terzioglu O. (2015). Clinical and genetic analysis of pediatric patients with Wilson disease. Turk. J. Gastroenterol..

